# In Patients Over 50 Years, Increased Age Is Associated With Decreased Odds of Documented Loss of Consciousness After a Concussion

**DOI:** 10.3389/fneur.2020.00039

**Published:** 2020-01-31

**Authors:** Alessandro Orlando, Benjamin Rubin, Ripul Panchal, Allen Tanner, John Hudson, Kyle Harken, Robert Madayag, Gina Berg, David Bar-Or

**Affiliations:** ^1^Department of Trauma Research, Swedish Medical Center, Englewood, CO, United States; ^2^Department of Trauma Research, Penrose Hospital, Colorado Springs, CO, United States; ^3^Department of Trauma Research, Medical City Plano, Plano, TX, United States; ^4^Department of Trauma Research, St. Anthony Hospital, Lakewood, CO, United States; ^5^Department of Trauma Research, Research Medical Center, Kansas City, MO, United States; ^6^Department of Trauma Research, Wesley Medical Center, Wichita, KS, United States; ^7^Department of Neurosurgery, Swedish Medical Center, Englewood, CO, United States; ^8^Department of Neurosurgery, Medical City Plano, Plano, TX, United States; ^9^Department of Trauma Services, Penrose Hospital, Colorado Springs, CO, United States; ^10^Department of Neurosurgery, St. Anthony Hospital, Lakewood, CO, United States; ^11^Department of Trauma Services, Research Medical Center, Kansas City, MO, United States; ^12^Department of Trauma Services, St. Anthony Hospital, Lakewood, CO, United States

**Keywords:** concussion, loss of consciousness, age, mild, traumatic brain injury, national trauma data bank

## Abstract

**Background:** Advanced aged adults have the highest rate of traumatic brain injury (TBI) related hospital admissions, compared to younger age groups. Data were published in 2014 indicating differential injury and neurological responses to a TBI by age categories. In a recent article examining patients with mTBI and isolated subdural hematoma, it was found that older patients had a decreased risk of documented loss of consciousness (LOC). The primary objective was to determine the extent to which the odds of documented LOC changes with increasing age in a population of older adults suffering an isolated concussion and uncomplicated mTBI.

**Methods:** This was a retrospective study utilizing 6 years (2010–2015) of National Trauma Data Bank data. This study included patients with (1) diagnosis of concussion; (2) positive or negative loss of consciousness; (3) loss of consciousness durations no longer than 59 min or undefined; (4) age ≥50 years; (5) had a “fall” mechanism of injury; and (6) a valid emergency department Glasgow coma scale 13–15. We excluded patients (1) with any intracranial hemorrhage or intracranial injury of other and unspecified nature; (2) skull fracture; (3) an injury severity scale score >17; (4) a concussion with “unspecified” LOC (ICD-9: 850.9).

**Results:** There were 7,466 patients included in the study; the median (IQR) age was 70 (60–80) years. The risk of documented LOC was 71% (*n* = 5,319). An 80-year-old had 72% decreased odds of having a documented LOC, compared to a 50-year-old (OR = 0.28, 99.5%CI [0.23–0.34], *P* < 0.001). This association held when controlling for multiple demographic, comorbid, and clinical variables, and in sensitivity analyses.

**Conclusion:** These nationwide data suggest that in patients aged ≥50 years, a significant inverse association exists between age and odds of documented LOC after sustaining a fall-related concussion. Additional studies are needed to validate these findings and to investigate the triad of age, documented LOC, and intracranial hemorrhage. Clinical diagnostic criteria relying on LOC might be at risk of being modified by the association between increasing age and decreasing odds of LOC.

## Introduction

The aging landscape in the United States is changing and will bring with it changes in volume of certain injury types. According to the United States Census Bureau, in the next 16 years there will be more people >65 years old than younger than 18 years (1). Currently, advanced aged adults have the highest rate of traumatic brain injury (TBI) related hospital admissions compared to younger age groups (2). Data were published in 2014 indicating differential injury and neurological responses to a TBI by age categories (3). In this 2014 study by Salottolo et al., there were higher proportions of patients ≥65 years who presented with mild neurological symptoms, compared to those <65 years, independent of the anatomic severity of head injury. This finding highlighted the differences in post-TBI symptoms by age. As such, it is vital to continue to explore and describe differences in how increasing age impact signs and symptoms after sustaining a TBI.

One important aspect in which to investigate differences in age is in the reporting of loss of consciousness (LOC) following a mild TBI (mTBI). The rate of documented LOC after mTBI varies from 40% to 70%. Across nearly 2,500 patients with a Glasgow coma scale (GCS) of 15, the risk of documented LOC was 69% (4). Similarly, in a population of 210 blast-related TBIs, the risk of documented LOC was 68% (5). In other studies examining mild to moderate TBI, however, the risk of documented LOC was lower (40–55%) (6–9). Whereas in a Colorado population-based survey of nearly 450,000 residents, the lifetime prevalence of LOC after mTBI was ~50% (10). It is clear that documented LOC following an mTBI is a frequent occurrence and likely a direct result of the accelerative, decelerative, rotational, and blunt forces incurred by the brain during injury.

In a recent article examining patients with mTBI and isolated subdural hematoma, it was found that older patients had a significantly decreased odds of documented LOC (6). This finding highlighted an important relationship worth understanding: Does a patient's age change the odds of LOC after a head injury? Thus, the primary objective of the current study was to determine the extent to which the odds of documented LOC changes with increasing age in a population of older adults suffering an isolated concussion and uncomplicated mTBI. Furthermore, since increasing age is associated with increased volume of cerebrospinal fluid (CSF) (11–15), this dynamic could modify the effect between increasing age and documented LOC. Thus, the secondary objective was to investigate if mild GCS scores of 13, 14, and 15 modify the effect between odds of documented LOC and age.

## Materials and Methods

### Study Site and Patient Population

This study was conducted using de-identified data from the National Trauma Data Bank (NTDB). The NTDB is a publicly available repository of data collected on trauma patients across the USA and removed of all protected health information; more information about the NTDB can be found at https://www.facs.org/quality-programs/trauma/ntdb. The inclusion criteria were as follows: (1) included in the NTDB datasets 2010–2015; (2) age ≥50 years; (3) diagnosis of concussion with documented LOC (ICD-9: 850.00–850.5); (4) a documented LOC no longer than 59 min, “brief,” or an undefined length of time; (5) had a mechanism of injury recorded as a fall from same level, or a fall on or from stairs or steps; and (6) an ED admission GCS 13–15. Patients were excluded for (1) presenting with any intracranial laceration, contusion, or hemorrhage (ICD-9: 851–853.19); (2) presenting with an intracranial injury of other and unspecified nature (ICD-9: 854–854.19, e.g., diffuse axonal injury); (3) sustaining a skull fracture (ICD-9: 800–804.99); (4) an injury severity score (ISS) >17; (5) an invalid admission ED GCS score (e.g., due to intubation, sedation, or injury); (6) a concussion with an unspecified LOC (ICD-9 850.9). The last exclusion criterion removed all patients in whom the presence or absence of LOC was unknown.

Different mechanisms of injury (e.g., motor vehicle accident vs. sports injury vs. ground-level fall) can result in different forces placed on the brain and different severities of TBI. Additionally, there is likely a different distribution of mechanisms of injury across ages; for example, young patients are likely to suffer a concussion due to a motor vehicle accident, whereas older patients are likely to suffer a concussion to a fall. These differences would likely confound the relationships being explored in this study. Attempting to control for this confounding factor, we limited our study population to only those patients who suffered a TBI from a fall from the same height or a fall from stairs or steps, as recorded in the NTDB. Furthermore, we excluded patients who presented with ICH because of the likely confounding interaction between age-related cerebral atrophy, risk of ICH, and risk of documented LOC. For example, a minor head injury might tear a blood vessel in an older patient, causing a subdural hematoma; however, the impact might not be significant enough to cause a documented LOC. On the other hand, the impact force required to cause a subdural hematoma in a younger patient is higher than their older counterpart, and would likely carry a higher risk of suffering a documented LOC. Similarly, patients sustaining a skull fracture were excluded because a skull fracture is indicative of a high-velocity impact to the skull; this study aimed to study documented LOC in the setting of low-velocity impacts to the skull.

Lastly, an ISS >17 was used as an exclusion criterion for this study because it was a proxy for isolated head injuries. Ideally, individual abbreviated injury scale (AIS) scores—which are used to calculate the ISS—are used to identify isolated head injuries; however, individual AIS scores are not consistently entered into the NTDB because they are not required. On the other hand, ISS scores are consistently entered into the NTDB. Because concussion AIS scores can range from 1 to 3, and because isolated head injuries can be defined as not having AIS scores higher than two for any injury area other than the head, the maximum ISS score a patient can have with an isolated concussion is 17. This study was not considered human subjects research because (1) the data are publicly available through the American College of Surgeons, (2) the data were not collected specifically for this study, and (3) all data were provided with all identifying information removed.

### Outcomes and Covariates

The primary exposure variable for this study was age, analyzed as a continuous variable. The primary outcome variable was documented LOC (positive or negative), and the secondary outcomes were hospital length of stay (HLOS), in-hospital mortality, and hospital discharge disposition. Covariates analyzed for this study included the following: age, sex (male vs. female), race (black or other vs. white), ethnicity (Hispanic or Latino vs. not Hispanic or Latino), comorbid conditions (hypertension requiring medication, diabetes mellitus, congestive heart failure, current smoker, history of myocardial infarction, and history of peripheral vascular disease, dementia), emergency department GCS (ED GCS, 13, 14, 15), length of documented LOC (obtained from ICD-9 code), inter-hospital transfer (y/n), normal ED oxygenation (95–100%), normal ED systolic blood pressure (90 to <120 mmHg), normal ED body temperature (36.4–37.6°C), normal ED pulse (60–100 beats/min), normal ED respiratory rate (12–16 breaths/min), payment status (Medicaid or Medicare, private, self-pay, not billed, and other), and hospital length of stay (HLOS, days).

### Statistical Analysis

Univariate analyses utilized χ^2^ or Fisher exact test to compare proportions, while Student's *t*-test or Wilcoxon Ranked Sum test compared continuous variables between study groups; test selection was dependent on the normality of the distribution. The Cochrane-Armitage trend test was used to examine trends between age deciles and proportions of documented LOC; *p*-values for this test are shown as “*P*-trend.” Generalized linear models were used to examine differences in the mean ISS value across age deciles, and to examine the interaction effect of documented LOC on ISS and age decile. The primary hypothesis was examined using a multivariable stepwise logistic regression model was used to compare the odds of documented LOC by age (continuous). Four models were built to estimate the odds ratio for recorded loss of consciousness. The first model shows the unadjusted relationship between age and recorded LOC; model 2 added demographic characteristics that were significantly different between recorded LOC groups; model 3 added pre-injury comorbidities that were significantly different between LOC groups; finally, model 4 added clinical characteristics that were significantly different between groups. To identify the best fitting model, a fifth model was created using a stepwise selection method with entry and exit criteria of 0.005; all variables in model 4 were available for selection into model 5; all two-way interactions were tested for all final variables selected into model 5. Hosmer-Lemeshow goodness-of-fit test *p*-values assessed how well each logistic regression model fit the data; *P*-values < 0.05 indicated poor fit. Furthermore, a logistic regression model compared the odds of having an HLOS >2 days between documented LOC groups; this model was adjusted for payment status. In-hospital mortality was examined via a stepwise multivariable logistic regression model, with documented LOC and age available for inclusion in the model. Additionally, the hospital discharge disposition analysis excluded patients who suffered in-hospital mortality. Increasing the threshold for statistical significance has been suggested in an effort to reduce the high number of irreproducible clinical studies (16). Therefore, all tests were two-tailed with an alpha value of 0.005, with 99.5% confidence intervals reported. SAS 9.4 (Cary, NC) was used for all analyses.

## Results

**Figure 1:**
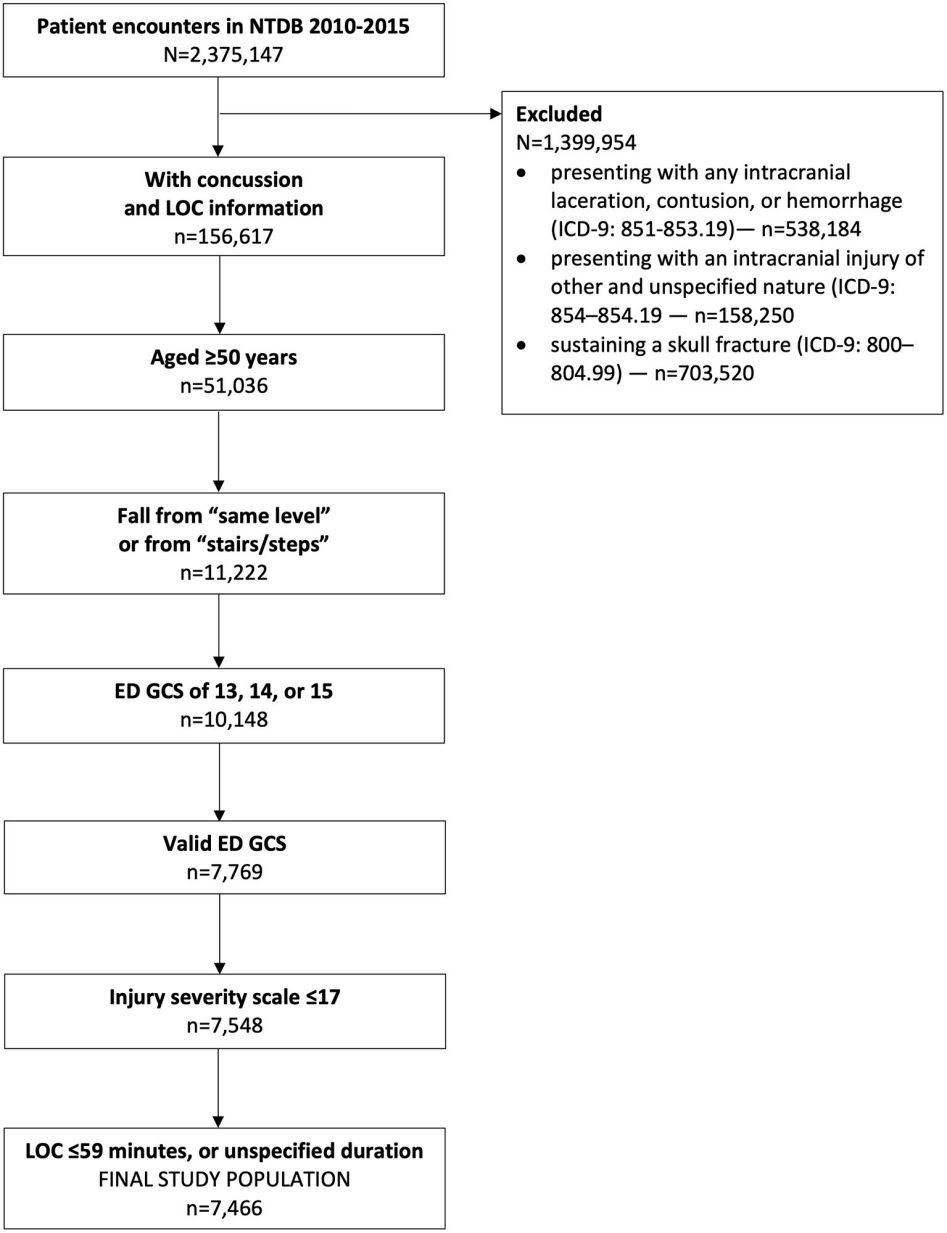
Flow diagram for study inclusion.

This study included 7,466 patients (Figure 1). The median age was 70 (60–80) years, and a slight majority of patients were females (Table 1). A significantly smaller proportion of patients reporting a LOC had hypertension requiring medication and congestive heart failure but had a significantly higher proportion who were current smokers. Most concussions were associated with a GCS 15; only 187 (2.5%) had a GCS 13. Many patients presented to the ED with abnormal systolic blood pressure and respiratory rate, but with normal oxygen, body temperature, and pulse vital signs. The overall median (IQR) ISS score was 5 (2–9), and ~12% of patients were transferred from another facility.

**Table 1:** Patient demographics and clinical characteristics by loss of consciousness.

**Variable, *n* (%)**	**No documented LOC *n* = 2,147**	**Documented LOC *n* = 5,319**	***P***
Age			
Mean (SD)	73.9 (10.9)	68.4 (11.4)	<0.001
Median (IQR)	76 (66–83)	68 (58–78)	<0.001
50–59	305 (14.2%)	1,500 (28.2%)	<0.001
60–69	389 (18.2%)	1,363 (25.6%)	
70–79	634 (29.5%)	1,269 (23.9%)	
≥80	819 (38.2%)	1,187 (22.3%)	
Sex			<0.001
Female	1,293 (60.2%)	2,485 (46.7%)	
Male	854 (39.8%)	2,838 (53.2%)	
Race			<0.001
Black or other	361 (16.8%)	1,104 (20.8%)	
White	1,786 (83.2%)	4,215 (79.2%)	
Ethnicity			0.05
Hispanic or Latino	103 (5.9%)	340 (7.3%)	
Not Hispanic or Latino	1,645 (94.1%)	4,309 (92.7%)	
Comorbid conditions			
Hypertension requiring medication	1,340 (69.6%)	2,977 (62.9%)	<0.001
Diabetes mellitus	538 (30.0%)	1,202 (25.4%)	0.03
Congestive heart failure	184 (9.6%)	293 (6.2%)	<0.001
Current smoker	177 (9.2%)	780 (16.5%)	<0.001
History of myocardial infarction	49 (2.6%)	175 (3.7%)	0.02
History of peripheral vascular disease	20 (1.0%)	46 (1.0%)	0.80
Dementia	173 (9.0%)	239 (5.1%)	<0.001
Injury severity scale			
Mean (SD)	4.8 (3.9)	7.4 (4.0)	<0.001
Median (IQR)	3 (2–6)	5 (5–9)	<0.001
ED GCS			<0.001
13	41 (1.9%)	146 (2.7%)	
14	242 (11.3%)	960 (18.1%)	
15	1,864 (86.8%)	4,213 (79.2%)	
Length of LOC			
Brief	–	143 (2.9%)	
≤30 min	–	2,097 (39.4%)	
31–59 min	–	24 (0.5%)	
Unspecified duration	–	3,055 (57.4%)	
Inter-hospital transfer	244 (11.4%)	679 (12.8%)	0.10
ED vital signs			
Normal oxygen	1,697 (85.1%)	4,203 (86.8%)	0.06
Normal systolic blood pressure	255 (11.9%)	767 (14.5%)	0.004
Normal body temperature	1,461 (68.1%)	3,500 (65.8%)	0.06
Normal pulse	1,772 (82.8%)	4,238 (80.0%)	0.006
Normal respiratory rate	648 (30.4%)	1,586 (30.1%)	0.80

*n = 7,466. LOC, loss of consciousness; SD, standard deviation; IQR, interquartile range; ED, emergency department; GCS, Glasgow coma scale. Not all counts add to group totals due to missing values*.

**Figure 2:**
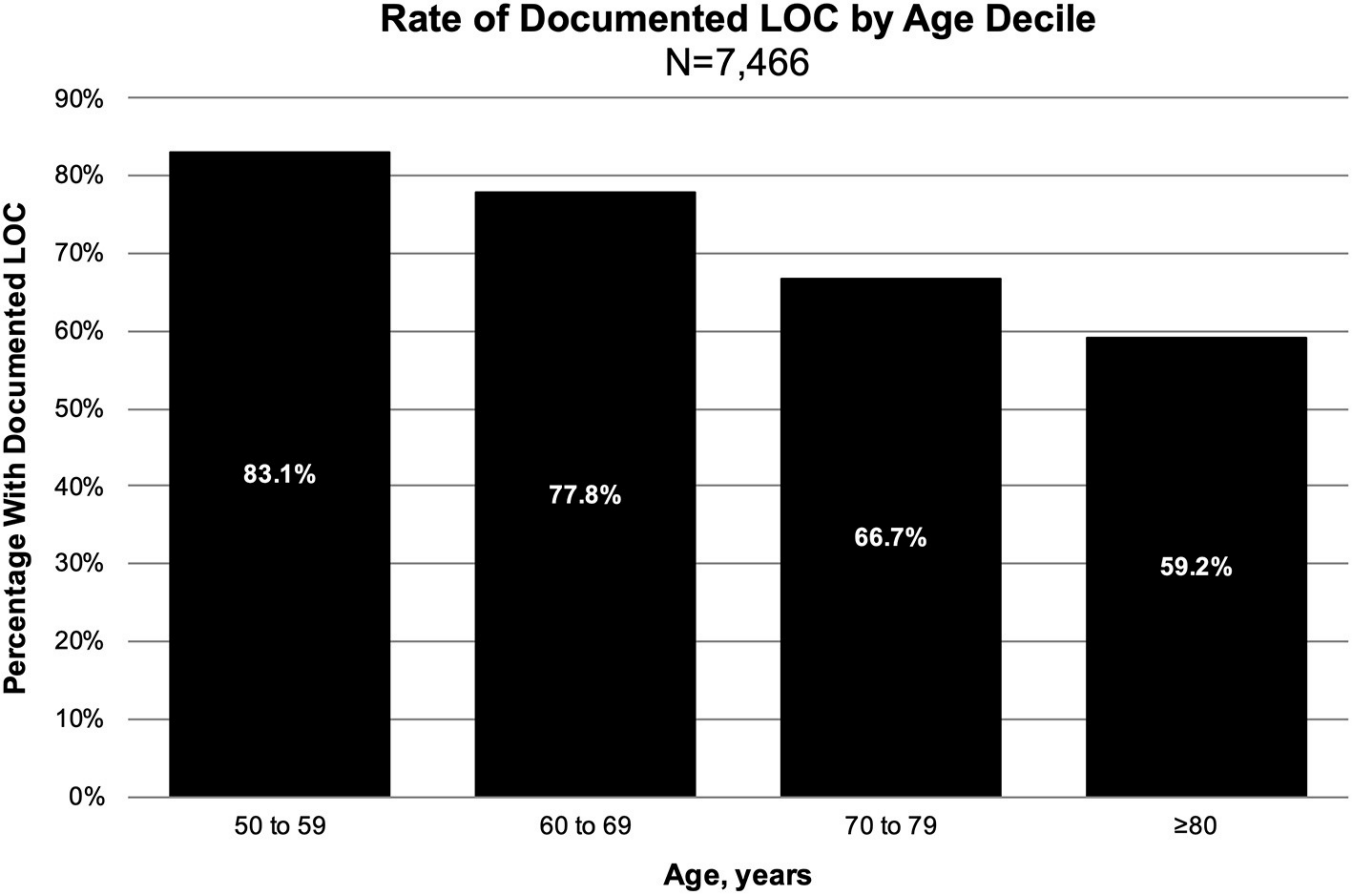
Risk of loss of consciousness by age decile.

The overall proportion of documented LOC was 71% (*n* = 5,319). A majority of documented LOC episodes had an “unspecified duration” or a duration of ≤30 min (Table 1). There was a significantly higher proportion of males in the documented LOC group compared to the no documented LOC group. Furthermore, there was a significant trend between increasing age decile and decreasing risk of documented LOC (*P*-trend < 0.001, Figure 2), and between decreasing GCS score and increasing risk of documented LOC (*P*-trend < 0.001); the probability of documented LOC in GCS 15, 14, and 13 was 69, 80, and 78%.

**Figure 3:**
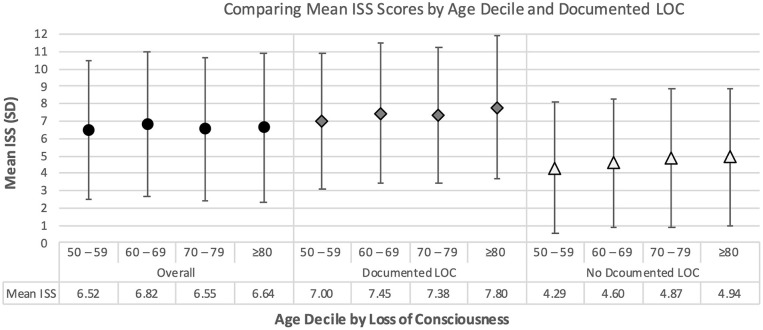
Mean injury severity scale scores by age decile and documented loss of consciousness.

These study data suggest there was no significant difference in the mean ISS score across all age deciles (Figure 3, *F* = 1.90, *P* = 0.13). Although patients with documented LOC have higher AIS scores–and thus higher ISS scores–than patients without documented LOC, documented LOC status did not significantly change the trends in ISS across each age decile (Figure 3, *P*-interaction = 0.55).

**Figure 4:**
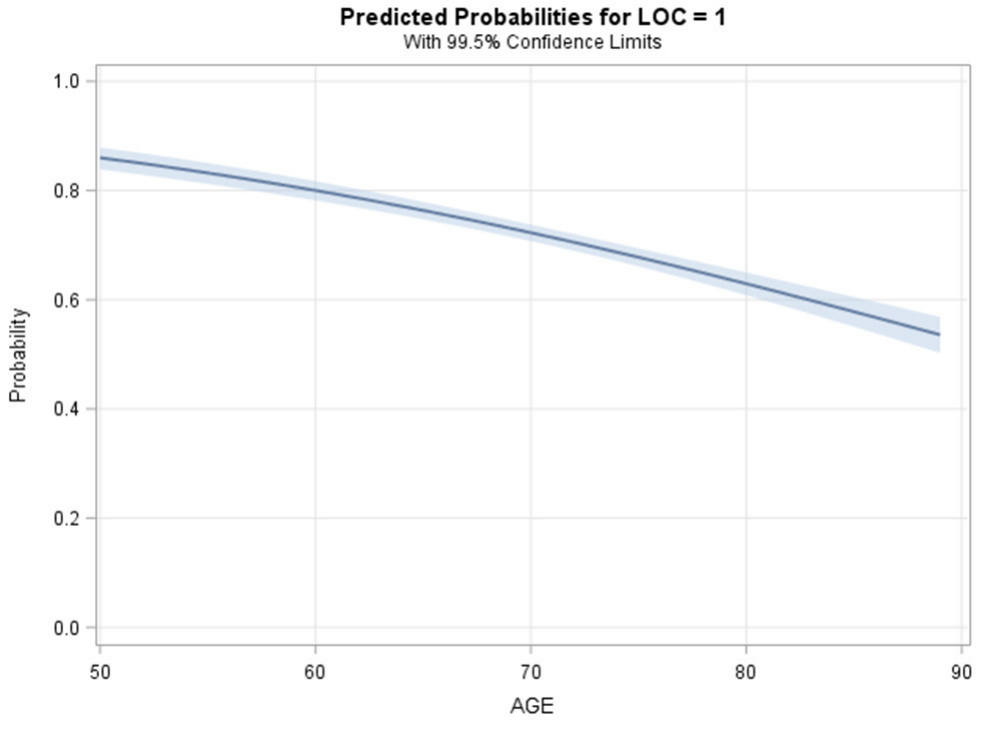
Modeled probability of documented loss of consciousness by age.

All logistic regression models supported an inverse association between age and odds of documented LOC (Table 2). The unadjusted logistic regression model (Model 1) analyzing age (continuous) and documented LOC suggested that every 1-year increase in age beyond 50 was significantly associated with a 4.2% decrease in the odds of documented LOC (Figure 4, OR = 0.96, 99.5%CI [0.95–0.96], *P* < 0.001, Goodness-of-fit *P* = 0.43). Compared to a 50-years-old patient, an 80-years-old patient had 72% decreased odds of documented LOC after suffering an isolated concussion (OR = 0.28, 99.5%CI [0.23–0.34], *P* < 0.001). Across logistic regression models 2–5, there was a consistent relationship between increasing age and decreasing odds of recorded LOC, but model fit was lacking. This indicated that covariates across demographic, comorbid, and clinical characteristics only minimally attenuated the unadjusted relationship between increasing age and recorded LOC (Table 2). The final model, model 5, contained five variables: age (continuous), sex, gender, dementia, and ED GCS; this model fit the data well (Goodness-of-fit *P* = 0.23). After adjustment, each 10-year increase in age was associated with a 32% reduction in the odds of documented LOC after a concussion (OR = 0.68, 99.5%CI [0.63–0.73]), *P* < 0.001). There were no significant two-way interactions between any of the covariates in model 5. Specifically, ED GCS was found to not significantly modify the effect between age and documented LOC in model 5 (interaction *P* = 0.46).

**Table 2:** Models estimating odds ratios for documented loss of consciousness.

**OR (99.5% CI)**	**Model 1**	**Model 2**	**Model 3**	**Model 4**	**Model 5**
Age (every 10-year increase)	0.65 (0.61, 0.70)	0.67 (0.62, 0.71)	0.70 (0.65, 0.76)	0.70 (0.65, 0.76)	0.68 (0.63, 0.73)
Female sex		0.65 (0.56, 0.76)	0.65 (0.56, 0.77)	0.66 (0.56, 0.78)	0.67 (0.56, 0.77)
Race					
White		Ref.	Ref.	Ref.	Ref.
Black		0.89 (0.69, 1.14)	0.95 (0.73, 1.24)	0.95 (0.72, 1.24)	0.93 (0.72, 1.22)
Other		1.39 (1.05, 1.83)	1.48 (1.09, 1.99)	1.42 (1.05, 1.93)	1.41 (1.04, 1.90)
Hypertension requiring medication			0.91 (0.77, 1.08)	0.93 (0.78, 1.10)	
Congestive heart failure			0.76 (0.57, 1.00)	0.75 (0.57, 1.00)	
Current smoker			1.28 (0.98, 1.66)	1.25 (0.96, 1.62)	
Dementia			0.80 (0.59, 1.09)	0.72 (0.53, 0.99)	0.73 (0.54, 0.99)
ED Glasgow coma scale					
13				Ref.	Ref.
14				1.27 (0.71, 2.26)	1.23 (0.69, 2.20)
15				0.70 (0.41, 1.21)	0.68 (0.39, 1.17)
Normal systolic blood pressure				1.13 (0.89, 1.43)	
Hosmer-Lemeshow Goodness-of-Fit	0.43	0.04	0.07	0.07	0.23

*OR, odds ratio; CI, confidence interval; ED, emergency department*.

The overall median (IQR) HLOS was 3 (1–5) days, and there was no significant difference in median HLOS between study groups (Table 3); 17 patients were missing HLOS data. After adjusting for payment status, patients suffering a documented LOC did not have significantly increased odds of having an HLOS >2 days, compared to patients not suffering a documented LOC (OR = 1.15, 99.5%CI [0.99–1.33], *P* = 0.01, Goodness-of-fit *P* = 0.97). The overall risk of in-hospital mortality was 0.8% (*n* = 57). According to the stepwise logistic regression model, age was a significant predictor of in-hospital mortality, whereas documented LOC was not. Each 10-year increase in age beyond year 50 carried a 93% increase in the odds of dying in the hospital (OR = 1.93, 99.5%CI [1.31–2.84], *P* < 0.001, Goodness-of-fit *P* = 0.18). It must be noted that the overall probability of in-hospital mortality was <5% for a 90-year-old. Furthermore, there were significant differences between documented LOC groups in hospital discharge disposition (Table 3). Patients who suffered a documented LOC had a higher proportion discharged to home/home health, compared to patients who did not suffer a documented LOC.

**Table 3:** Patient outcomes by documented loss of consciousness.

**Variable, No. (%)**	**No documented LOC *n* = 2,147 (28.8%)**	**Documented LOC *n* = 5,319 (71.2%)**	***P***
Hospital LOS, median (IQR)	2 (1–5)	3 (1–5)	0.06
Hospital discharge disposition^a^			<0.001
Acute care	48 (2.7%)	135 (2.9%)	
Against medical advice	10 (0.6%)	52 (1.1%)	
Home/home health	1,190 (65.8%)	3,275 (70.7%)	
Nursing home	380 (21.0%)	701 (15.1%)	
Rehabilitation	172 (9.5%)	426 (9.2%)	
Hospice	10 (0.6%)	22 (0.5%)	
Other	9 (0.5%)	43 (0.9%)	
In-hospital mortality, *n* (%)	16 (0.8%)	41 (0.8%)	0.91

*n = 7,466*.

*LOC, loss of consciousness; LOS, length of stay; IQR, interquartile range*.

a *993 patients missing hospital discharge disposition: 328 No documented LOC, 665 Documented LOC*.

Four several sensitivity tests were conducted to assess for potential bias and confounding; all sensitivity analyses included the appropriate variables from the final logistic regression model. First, all patients with a documented LOC with a duration of “unspecified duration” were excluded (*n* = 3,044). The primary outcome results remained consistent with the study results: for each 10-year increase in age beyond 50 years, the odds of LOC decreased by 35% (OR = 0.65, 99.5%CI [0.60, 0.71]). Second, the primary analysis was stratified by each GCS score (13, 14, or 15), instead of including GCS as a covariate; these results mirrored the primary results. The odds of LOC for each 10-year increase in age beyond 50 years were as follows: GCS 13 (OR = 0.57, 99.5%CI [0.36, 0.90]); GCS 14 (OR = 0.61, 99.5%CI [0.50, 0.74]); and GCS 15 (OR = 0.68, 99.5%CI [0.63, 0.73]). Third, the primary analysis was stratified by fall type (same-level vs. stairs or steps); these results continued to mirror the primary results. For each 10-year increase in age, the odds of LOC were 0.66 (99.5%CI [0.61, 0.72]) for patients with same-level falls, and were 0.72 (99.5%CI [0.64, 0.81]) for patients with falls from stairs or steps. Lastly, all patients with a preexisting comorbidity of dementia were excluded (*n* = 412). Yet, the results were no different from the primary analysis: in patients without dementia at hospital admission, the odds of documented LOC decreased by 32% for every 10-year increase in age (99.5%CI [0.63, 0.73]).

## Discussion

This is the first study to document an association between age and documented LOC, in the setting of isolated concussions. Although this study is unique, it is similar to other published studies (4, 8, 9, 17, 18). These data showed a significant relationship between increasing age and the probability of documented LOC, such that the oldest patients had the lowest odds of documented LOC, and the youngest patients had the highest odds. Furthermore, we failed to reject the null hypothesis regarding the interaction between GCS and age on documented LOC; the effect of age on odds of documented LOC was consistent across mild GCS scores, and sex. Finally, the odds of documented LOC were not associated with hospital LOS or in-hospital mortality. The results from these secondary outcomes analyses support this population as one suffering a low-impact injury in the setting of an isolated concussion.

Although causality cannot be established with these retrospective data, age might exert its effect on documented LOC via the increased volume of CSF and decreased cerebral volume. This is supported by multiple imaging studies observing that the volume of CSF increases with age (11–15). Gur et al. examined age-related cerebral atrophy in 69 volunteers aged 18–80 years using magnetic resonance imaging (MRI) (12). Their data suggested that brain volume significantly decreased with increasing age, and was simultaneous with significant increases in CSF volume. However, it is important to note the possibility for non-CSF age-related processes to explain the observed associations between age and documented LOC. Published data have suggested that increased intracranial pressure (ICP) can be protective against white matter changes following head trauma (19–21). Thus, it is possible that older individuals have higher pre-injury ICP than younger patients, helping to protect the elderly from LOC. However, a recent small study by Pedersen et al. suggested a lack of significant difference in 24-h ICP values between children (range 4–17 years) and adults (range 18–85 years) (22). Therefore, it is unlikely that increased ICP is contributing to a lower odds of documented LOC in elderly patients. Nevertheless, further research is necessary to investigate and explore possible mechanisms underlying the observed relationship between increased age and decreased risk of LOC.

The data from the current study are consistent with two hypotheses for what causes alterations in consciousness following mTBI: the convulsive, and the centripetal hypotheses. The convulsive hypothesis states that the mechanical forces on the brain resulting from a TBI cause electrical activity akin to seizures or electroconvulsive therapy. Meanwhile, the centripetal hypothesis states that the rotational and linear forces experienced by the cortex during a head injury cause shearing strains and stresses, resulting in the disconnection of nerve fibers (23). These disconnected nerve fibers are posited to affect consciousness negatively. Although the mechanism by which a head injury causes a documented LOC is likely a combination of the centripetal and convulsive hypotheses, and potentially others, it is possible that age-related cortical and subcortical changes could mitigate these pathways. Because the current study excluded patients with diffuse axonal injury, the centripetal hypothesis is likely not to explain the documented LOC seen in the current study.

When these results are placed into the context of existing TBI literature, a lack of documented LOC might be a poor indicator of a lack of intracranial injury in older adults. In a 2014 study of TBIs in the *Journal of the American Medical Association*, the neurological scale, GCS, was compared to the anatomical injury scale, AIS (3). Those data indicated that independent of the anatomic severity of TBI (e.g., size of hemorrhage), older patients displayed fewer neurological deficits. A potential explanation for this observation advanced by the authors was that age-related cerebral atrophy (higher volumes of CSF) allowed for a higher volume of hemorrhage before affecting neurological systems. Unfortunately, multiple decision criteria (New Orleans Criteria, Canadian CT Head Rule) identifying which patients are at risk of having an ICH after TBI have excluded patients without documented LOC (24, 25). This exclusion criterion removes 30 to 50% of all TBI patients, a large proportion of which might be at risk of having a symptom-free ICH due to age-related cerebral atrophy (4–9). There is a need for more research investigating if the risk of an in-hospital delayed ICH is modified by the presence or absence of documented LOC following a mTBI.

This study has several limitations. First, it utilized retrospective data from the NTDB. Although the NTDB implements multiple data checks when participating hospitals upload data, it relies on each hospital to upload accurate and complete data. The primary outcome of this study was *documented LOC*; as such, it was impossible to independently confirm the presence or absence of LOC in this study population. Furthermore, because not all data points were required for the NTDB, we were unable to define isolated TBI according to individual AIS scores and body regions (e.g., no AIS score >2 in any body region except the head). Also, we assumed that durations of documented LOC that were unspecified were of shorter durations (e.g., ≤30 min), not longer; however, sensitivity analyses excluding all LOCs with “unspecified durations” yielded similar significant results to the main analysis.

Moreover, all hospital admission-based studies have an inherent possibility for selection bias because they are limited to those who present to the hospital. This study had the potential to suffer from selection bias resulting from differential admission rates by patient age and injury severity. It was posited that younger adults suffering from concussion and mTBI would present more often with higher ISS scores, because those with lower ISS scores would not seek medical attention. Conversely, it was expected that older adults would present more often with lower ISS scores because the threshold for admission is lower in this age group. Through the analysis of admission ISS scores by age decile and documented LOC status, the current study results suggest that patients of all ages >50 years were similarly injured vis-à-vis ISS scores, and that the main finding of age being associated with reduced odds of documented LOC is likely to reflect age-related cranial changes, not an epi-phenomenon of the interplay between age, injury severity, and likelihood of hospital admission. Also, sensitivity analyses stratifying separately by GCS and fall type resulted in similar associations between increasing age and decreasing odds of documented LOC. This finding was suggestive of similar injury severities across age categories. These sensitivity analyses buttressed the positive impact of the inclusion and exclusion criteria on minimizing potential biases and confounding variables.

It could also be argued that the presence of dementia (which increases in prevalence with increasing age) could result in the differential ascertainment of post-concussion LOC and thus introduce differential misclassification bias, such that older adults with dementia are classified as no documented LOC; this was investigated using a sensitivity analysis. This analysis excluded all patients with dementia at hospital admission and saw a similar relationship between increasing age and decreasing odds of documented LOC. Therefore, the presence of dementia on admission is not likely to affect the effect between age and documented LOC in the current study. Notwithstanding the competing explanations for the study observations, and the potential for residual confounding, these data highlight the need to generate more information on how documented LOC impacts TBI symptoms and how it relates to short-term and long-term patient outcomes.

In conclusion, this large national data set suggested an inverse association between age and odds of documented LOC after a fall-related concussion. Older adult patients had significantly decreased odds of documented LOC after a concussion, compared to their younger counterparts. Future research should seek to assess how the relationship between the volume of CSF and risk of documented LOC changes with increasing age. More importantly, future research should examine the relationship between documented LOC and the risk of acute and delayed ICH. These lines of investigation have the potential to influence how documented LOC and patient age are incorporated into the management of mTBI and concussions. Clinical diagnostic criteria relying on LOC might be at risk of being modified by the association between increasing age and decreasing odds of LOC.

## Data Availability Statement

The datasets generated for this study are available on request to the corresponding author.

## Author Contributions

AO contributed to the conception and design of the study, acquisition, analysis, interpretation of data, drafting and revising of the manuscript, final approval of the manuscript version to be published, and agreeing to be accountable for all aspects of work. BR, RP, and AT contributed to the interpretation of data, revising of the manuscript, final approval of the manuscript version to be published, and agreeing to be accountable for all aspects of work. JH contributed to the analysis and interpretation of data, revising of the manuscript, final approval of the manuscript version to be published, and agreeing to be accountable for all aspects of work. KH and RM contributed to the interpretation of data, revising of the manuscript, final approval of the manuscript version to be published, and agreeing to be accountable for all aspects of work. GB contributed to the analysis and interpretation of data, revising of the manuscript, final approval of the manuscript version to be published, and agreeing to be accountable for all aspects of work. DB-O contributed to the design of the study, interpretation of data, revising of the manuscript, final approval of the manuscript version to be published, and agreeing to be accountable for all aspects of work.

### Conflict of Interest

The authors declare that the research was conducted in the absence of any commercial or financial relationships that could be construed as a potential conflict of interest.
